# Enhanced rectification effect in silver chalcogenide-based thermal diode by using precipitation/dissolution of Ag impurity across the structure phase transition

**DOI:** 10.1080/14686996.2025.2549674

**Published:** 2025-08-28

**Authors:** Keisuke Hirata, Yusuke Goto, Tsunehiro Takeuchi

**Affiliations:** a Graduate School of Engineering, Toyota Technological Institute, Nagoya, Japan; b Research Center for Smart Energy Technology of Toyota Technological Institute, Nagoya, Japan

**Keywords:** Silver chalcogenides, thermal conductivity, lattice thermal conductivity, electronic thermal conductivity, thermal diode, heat flow, material engineering

## Abstract

For developing high-performance composite-type thermal diodes, this study focuses on silver chalcogenides, which undergo structural phase transitions in the temperature range of 350 K to 473 K, accompanied by a significant stepwise change in thermal conductivity. Ag_2 + *x*_Te_0.9_S_0.1_ (*x* = 0, 0.01, 0.02, 0.025, 0.03, 0.035, 0.04, and 0.05) and Ag_2_S_1 – *y*_Se_*y*_ (*y* = 0.35, 0.375, 0.4, 0.425, and 0.45) samples were synthesized with precisely controlled compositions, and their temperature-dependent thermal conductivity across the phase transition was studied with the composition dependence. Ag_2_Te_0.9_S_0.1_ exhibits a stepwise decrease in thermal conductivity with transitioning from the low-temperature phase (LTP) to the high-temperature phase (HTP), and this behavior was further enhanced by adding excess Ag. The added silver precipitated in the LTP and dissolved into the HTP of Ag_2_Te_0.9_S_0.1_, resulting in a maximum thermal conductivity change (*κ*_LTP_ / *κ*_HTP_) of 2.7-fold with the phase transition at *x* = 0.025. On the other hand, the Ag_2_S_1 – *y*_Se_*y*_ samples exhibited a stepwise increase in thermal conductivity with transitioning from the LTP to the HTP, and the maximum thermal conductivity change of *κ*_HTP_ / *κ*_LTP_ = 5 was observed at *y* = 0.4. A composite thermal diode was fabricated using Ag_2.025_Te_0.9_S_0.1_ and Ag_2_S_0.6_Se_0.4_ with the length ratio of Ag_2.025_Te_0.9_S_0.1_: Ag_2_S_0.6_Se_0.4_ = 47:53 and, consequently, exhibited *TRR* = 3.3 when it was placed between heat reservoirs maintained at *T*_H_ = 412 K and *T*_L_ = 300 K. This *TRR* value is the largest ever reported for all-solid-state composite thermal diodes.

## IMPACT STATEMENT

This work achieves record-high thermal rectification ratio of 3.3 in all-solid-state composite thermal diodes by enhancing thermal conductivity changes through precipitation/dissolution of silver impurities across phase transitions in silver chalcogenides.

## GRAPHICAL ABSTRACT

**Figure:**
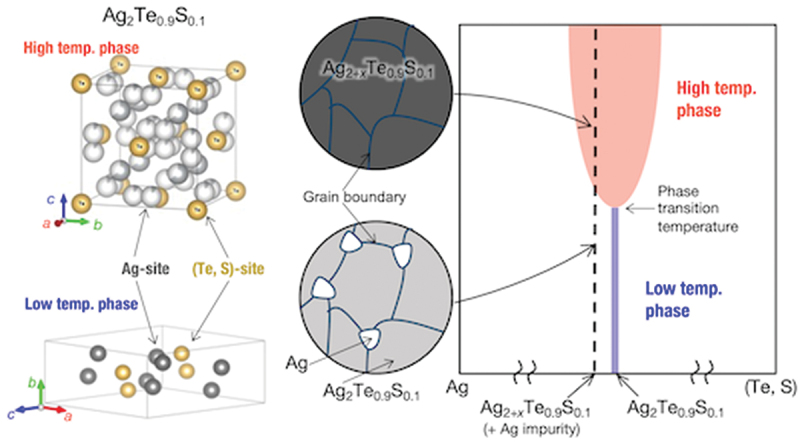

## Introduction

To achieve a sustainable, energy-efficient society, the effective utilization of unused waste heat has gained significant attention. This is considered one of the most important concepts in thermal management [1,2]. Establishing technologies for spatiotemporal heat flow control would enable us to efficiently transfer the heat to some places where heat energy is required. A thermal diode, which rectifies heat flow, is considered a key technology for thermal management, particularly when combined with magnetocaloric/electrocaloric refrigeration, thermal storage, and thermoelectric technologies [1,2].

**Figure 1.**
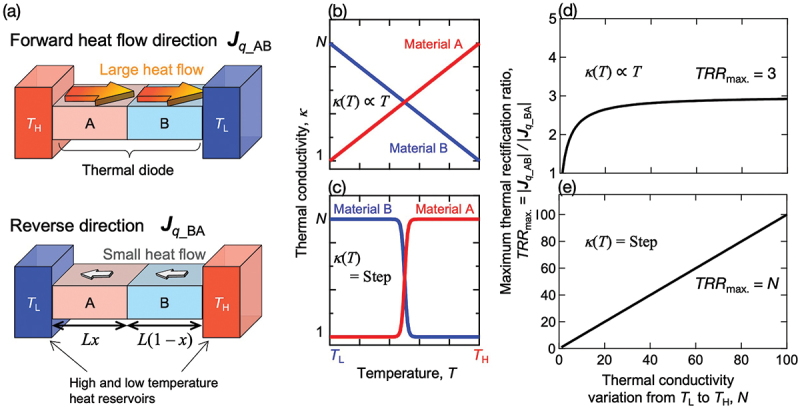
(a) schematic illustration of the working principle of a composite thermal diode. The composite thermal diode consists of hypothetical materials A and B possessing increasing and decreasing temperature-dependent thermal conductivity, respectively. It is placed between high- (*T*_H_) and low- (*T*_L_) temperature heat reservoirs [5,6]. a large heat flow |*J*_*q*_AB_| is obtainable in the forward heat flow direction, while a small heat flow |*J*_*q*_BA_| occurs in the reverse direction. Here, *L* and *x* represent the total length of the composite thermal diode and the length ratio of material a to B, respectively. (b, c) linear and stepwise temperature-dependent thermal conductivity of hypothetical materials A and B. The thermal conductivities of materials A and B are assumed to increase by a factor of *N* and decrease by a factor of 1/*N* from *T*_L_ to *T*_H_, respectively. (d, e) maximum thermal rectification ratio (*TRR*_Max._ = |*J*_*q*_AB_| / |*J*_*q*_BA_|) depending on the thermal conductivity variation *N*, for the cases of materials possessing linear and stepwise temperature-dependent thermal conductivity [7].

Several principles for realizing thermal rectification have been proposed to date [1,3,4]. Among these, we focus on solid-state thermal diodes that consist of two different materials with different temperature-dependent thermal conductivities [5,6]. As illustrated in Figure 1(a-c), a composite thermal diode consists of two materials: Material A, whose thermal conductivity *κ* increases with increasing temperature, and Material B, whose thermal conductivity decreases [5,6]. When Materials A and B are placed on the high-temperature side and the low-temperature side, respectively, both materials exhibit high thermal conductivity, resulting in a large heat flow magnitude |***J***_*q*_AB_|. On the other hand, when the positions of A and B are reversed, both materials exhibit low thermal conductivity, leading to a small heat flow magnitude |***J***_*q*_BA_|. The performance of a thermal diode is usually evaluated using the thermal rectification ratio (*TRR*), which is defined as the magnitude of the heat flow in the forward direction with the larger magnitude over that of the reverse direction of the smaller magnitude: *TRR* = |***J***_*q*_AB_| / |***J***_*q*_BA_|.

A large *TRR* requires materials with a significant temperature dependence of thermal conductivity. For example, as shown in Figure 1(b, d), the maximum *TRR* is limited to 3, when combining materials whose thermal conductivity is linearly increasing/decreasing with temperature. This limit is derived from a numerical calculation of macroscopic heat flow based on Fourier’s law and a hypothetical model assuming the conditions of no heat leak from the system, no interfacial thermal resistance, and steady state [8]. This limitation remains even if the thermal conductivity variation *N* (or 1/*N*) between *T*_L_ and *T*_H_ approaches infinity (or zero). The number of *TRR* = 3 does not contain any particularly fundamental physical significance and just provides a practical benchmark in device development since no all-solid-state composite thermal diode exceeding this value has been reported to date. When materials exhibiting a stepwise increase (*N*-fold) or decrease (1/*N*-fold) in thermal conductivity are employed, the upper limit of *TRR* is determined by the step size *N* of the thermal conductivity, as shown in Figure 1(c, e) [7]. So far, several reports have demonstrated thermal rectification effects using various materials across different temperature ranges [1,3,4]. In particular, phase-transition materials, which potentially exhibit stepwise changes in thermal conductivity, are considered one of the most promising candidates to realize large thermal rectification under a relatively small temperature difference. For instance, a composite thermal diode utilizing solid–liquid phase transition materials and polymers has been reported to show a relatively large *TRR* of more than 2 with a small temperature difference, ≤50 K [9–12]. Among them, the highest value, *TRR* = 3.5 (*T*_H_ = 323 K, *T*_L_ = 283 K), was achieved using a thermal diode consisting of calcium chloride hexahydrate possessing a solid-liquid phase transition and an aqueous solution of a thermos-responsive polymer, poly(N-isopropylacrylamide) [11]. Meanwhile, the highest *TRR* is still less than 3 for all-solid-state composite thermal diodes, which can be more easily integrated into mechanical structures for industrial applications if their constituent materials possess sufficient mechanical strength. The highest *TRR* of 2.7 (*T*_H_ = 405 K, *T*_L_ = 300 K) was observed in the silver-chalcogenides-based (Ag_2_S_0.6_Se_0.4_-Ag_2_Te_0.9_S_0.1_) thermal diode developed by our group [13]. Thus, new techniques to achieve *TRR* ≥3 are required for the development of all-solid-state thermal diodes.

**Figure 2.**
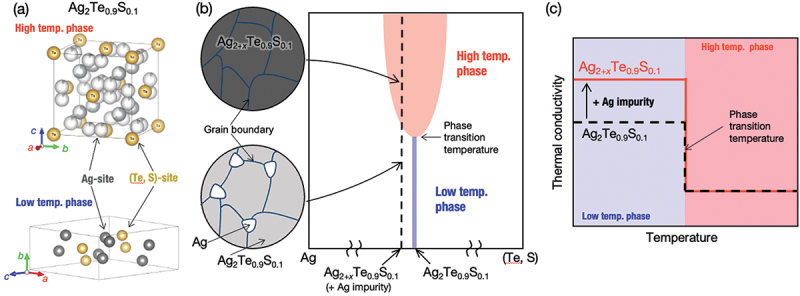
(a) crystal structures of low- and high-temperature phases of Ag_2_Te_0.9_S_0.1_ [17,18] drawn using VESTA [19]. The low-temperature phase has a monoclinic structure (*P*2_1_/*c* in the space group and *mP*12 in the Pearson symbol), while the high-temperature phase has a cubic structure (*Fm*-3*m* in the space group and *cF*12 in the Pearson symbol) [17,18]. in the high-temperature phase, several partly occupied Ag sites represent the average structure due to the nature of super Ag-ionic conduction. (b) a plausible phase diagram of the Ag-(S, Te) system and the microstructure of slightly Ag-added Ag_2+*x*_Te_0.9_S_0.1_. at the composition of Ag_2+*x*_Te_0.9_S_0.1_, Ag impurities precipitate in the low-temperature phase and dissolve into the matrix in the high-temperature phase, owing to differences in the single-phase region [20]. (c) schematic diagram of temperature-dependent thermal conductivities of Ag_2_Te_0.9_S_0.1_ [13] and Ag_2+*x*_Te_0.9_S_0.1_. The stepwise decrease in thermal conductivity across the phase transition is expected to be enhanced by the precipitation of high-thermal-conductivity Ag impurities [21] in the low-temperature phase.

In this study, we also aimed to enhance the performance of thermal diodes by employing the silver chalcogenides, Ag_2_Te_1–*x*_S_*x*_ and Ag_2_S_1–*y*_Se_*y*_, since they are used in the all-solid-state thermal diode that has achieved the highest *TRR* [13]. Typical silver chalcogenides, such as Ag₂S, Ag₂Se, and Ag₂Te, undergo structural phase transitions between *T*_*x*_ = 400 K and 450 K [14–16]. For example, as shown in Figure 2(a), their low-temperature phases (LTP) possess an ordered crystal structure, where Ag sites are fully occupied, with semiconducting electronic structure. Their high-temperature phases (HTP), on the other hand, exhibit significant disorder in the occupancy of Ag site due to super Ag-ionic conduction [17,18,22–25]. The phase transition temperature *T*_*x*_ can be controlled by partially substituting the chalcogen site [26]. Among the silver chalcogenides, Ag_2_Te_1–*x*_S_*x*_ (*x* ≤ 0.15) exhibits an Ag_2_Te-type crystal structure at room temperature. A stepwise decrease in thermal conductivity with a maximum reduction of about 0.5-fold is observable for Ag_2_Te_0.9_S_0.1_ with increasing temperature at around *T*_*x*_ [13]. A stepwise increase of thermal conductivity, on the other hand, is observable at around *T*_*x*_ with increasing temperature for Ag_2_S_1–*y*_Se_*y*_ (*y* ≤ 0.6). A maximum increase of 5-fold is achieved through a step-like behavior at *y* = 0.4 [13]. In both cases, the significant stepwise variations are attributed to the variation in electronic thermal conductivity in association with the carrier concentration change across the structural phase transition, as confirmed by electrical resistivity measurements and Wiedemann-Franz law analysis for silver chalcogenides [14,27,28]. An extremely low lattice thermal conductivity (~0.5 W m^–1^ K^−1^) both in their LTP and HTP [13,29] also contributes to the significant step-up and step-down behavior in the temperature dependence of thermal conductivity of Ag_2_S_0.6_Se_0.4_ and Ag_2_Te_0.9_S_0.1_, respectively, by reducing the unchanged background.

In our previous study achieving *TRR* = 2.7 with Ag_2_S_0.6_Se_0.4_-Ag_2_Te_0.9_S_0.1_ thermal diode, the following two reasons prevented us from observing *TRR* > 3: (1) the step-down thermal conductivity with increasing temperature observed in Ag_2_Te_0.9_S_0.1_ was relatively small compared with the step-up behavior in Ag_2_S_0.6_Se_0.4_; (2) there is insufficient data on the composition dependence of thermal conductivity in Ag_2_S_1–*y*_Se_*y*_ in the vicinity of *y* = 0.4, where the most significant step-up behavior was observed [13].

To enlarge the step-down ratio in thermal conductivity, we introduced a new strategy by considering the difference in the formation range of the LTP and the HTP. Although both the LTP and the HTP are displayed as a line compound in the phase diagram, the formation range of HTP is supposed to be wider than that of LTP because of the larger entropy effect [20,30]. Thus, an intentionally added tiny amount of Ag atoms precipitated in the LTP with a slightly silver-rich composition would dissolve into the HTP at high temperatures above *T*_*x*_ as schematically drawn in Figure 2(b). In such a case, the step-down behavior in thermal conductivity is supposed to be emphasized by the precipitated Ag atoms only in LTP owing to their high thermal conductivity (~400 Wm^−1^K^−1^ at room temperature [21]), as schematically shown in Figure 2(c). In addition, we decided to investigate the compositional dependence of the thermal conductivity of Ag_2_S_1–*y*_Se_*y*_ near *y* = 0.4 with a small composition step Δ*y* = 0.025, aiming to identify compositions where the step-up ratio in thermal conductivity shows the maximum value, which is not fully investigated in the previous study (Δ*y* = 0.1 or 0.2) [13,27].

In this study, therefore, we systematically prepared two series of silver chalcogenides at Ag_2+*x*_Te_0.9_S_0.1_ (*x* = 0, 0.01, 0.02, 0.025, 0.03, 0.035, 0.04, and 0.05) and Ag_2_S_1_-_*y*_Se_*y*_ (*y* = 0.35, 0.375, 0.4, 0.425, and 0.45) with precise control of their composition, and carefully measured their temperature dependence of thermal conductivity. Evaluating the composition dependence and temperature dependence of thermal conductivity across the structure phase transition, we succeeded in identifying the compositions, Ag_2.025_Te_0.9_S_0.1_ and Ag_2_S_0.6_Se_0.4_, where the maximum step-up and step-down ratios in thermal conductivity are observable, respectively. We fabricated the thermal diode using these materials and demonstrated that a maximum *TRR* of 3.3, the highest value ever reported for all-solid-state thermal diodes, is obtainable under conditions of *T*_H_ = 412 K and *T*_L_ = 300 K.

## Experimental procedure

Bulk samples were synthesized at the compositions of Ag_2_Te_0.9_S_0.1_ and Ag_2_S_1– *y*_Se_*y*_ (*y* = 0.35, 0.375, 0.4, 0.425, and 0.45) by a melting method. Pure powders of silver (purity of 3N), sulfur (4N), selenium (3N), and tellurium (3N) purchased from Kojundo Chemical Lab. (Japan) were weighed so as to be the stoichiometric ratios and mixed well using an agate mortar and pestle. The mixed powder was pressed into a cylindrical pellet at room temperature and sealed in an evacuated quartz tube (vacuum level: ~10 ^− 2^ Pa). The sealed tube was placed in a muffle furnace and heated to 1273 K for 8 hours, followed by a homogenization process at 1273 K for 12 hours, and then cooled down to 573 K in 8 hours, followed by furnace cooling to room temperature. The obtained slab was crushed into fine powder in an alumina mortar filled with liquid nitrogen. The additional silver powder (grain size: 75–150 µm) was mixed into the Ag_2_Te_0.9_S_0.1_ powder to prepare Ag_2 + *x*_Te_0.9_S_0.1_ samples with controlled silver content (*x* = 0, 0.01, 0.02, 0.025, 0.03, 0.035, 0.04, and 0.05). Based on the precision of the weighing method used in our sample preparation, the uncertainty in composition *x* in Ag_2+*x*_Te_0.9_S_0.1_ was estimated to be ± 0.001.

The hot-pressing method was employed to fabricate high-density bulk samples. The powder sample was placed in a carbon mold that was covered with carbon sheets and encircled by alumina powder. The sintering was conducted at a low vacuum pressure of less than 15 Pa at 573–673 K with a uniaxial pressure of 50 MPa for 15 minutes. The density of the obtained pellet samples, verified by the Archimedes method, exceeded 95% of the theoretical density.

The X-ray diffraction (XRD) with the Bragg-Brentano diffractometer and a Cu-*K*_*α*_ (wavelength:1.5418 Å) radiation source (Bruker D8 ADVANCE, U.S.A.) was employed to identify the phases involved in the synthesized Ag_2 + *x*_Te_0.9_S_0.1_ and Ag_2_S_1 – *y*_Se_*y*_ at room temperature. The Ag_2 + *x*_Te_0.9_S_0.1_ samples were measured in pellet form to verify the systematic variation of the Bragg peak intensities of (Ag)-precipitation due to the intentionally introduced excess amount of Ag. The Ag_2_S_1 – *y*_Se_*y*_ samples were measured in powder form to confirm the systematic lattice constant changes due to the substitution of S by Se.

Scanning electron microscopy (SEM) equipped with energy-dispersive X-ray spectroscopy (EDX) was performed using SU6600 (Hitachi, Japan) at an acceleration voltage of 20 keV.

The temperature dependence of thermal conductivity *κ*(*T*) was determined from the equation *κ*(*T*) = *ρ C_P_*(*T*) *d*(*T*). Here, *ρ* represents the sample density estimated by the Archimedes method at room temperature using XS205 (METTLER TOLEDO, U.S.A.); *d*(*T*) and *C_P_*(*T*) represent thermal diffusivity and specific heat capacity, respectively, obtained by the laser flash method using LFA457 (NETZSCH, Germany) [31,32]. The laser flash measurement was conducted using the pellet-shaped samples with ~10 mm in diameter and 1–2 mm in thickness under an Ar flow atmosphere. For each determined set-point, the measurement was conducted at least three times, and the data were averaged. Measurement error was estimated to be approximately 7%, for which the main factor is the sample size [29].

We calculated the most promising material combinations for thermal diodes potentially possessing the highest thermal rectification ratio from the obtained data on temperature- and composition-dependent thermal conductivity. The calculation follows a reported methodology based on the integral formulation of Fourier’s law [8], requiring information on the temperature range, temperature-dependent thermal conductivity, and length ratio of constituent materials in a composite thermal diode, as shown in Figure 1 and in the following equation:

$$\mathrm{TRR}=\frac{\left|J_{q\_\mathrm{AB}}\right|}{\left|J_{q{\_}_{\mathrm{BA}}}\right|}=\frac{\int_{T_{L}}^{T_{\mathrm{AB}}}\kappa_{B}(T)\mathrm{dT}+\int_{T_{\mathrm{AB}}}^{T_{H}}\kappa_{A}(T)\mathrm{dT}}{\int_{T_{L}}^{T_{\mathrm{BA}}}\kappa_{A}(T)\mathrm{dT}+\int_{T_{\mathrm{BA}}}^{T_{H}}\kappa_{A}(T)\mathrm{dT}}$$

where ***J***_*q*_AB_ and ***J***_*q*_BA_ represent heat flux for forward and reverse directions, respectively; *κ*_A_(*T*) and *κ*_B_(*T*) represent temperature-dependent thermal conductivity of hypothetical materials A and B, respectively; *L* is the total length of the composite thermal diode, and *x* is the length ratio of material A to *L*; *T*_H_ and *T*_L_ are the temperatures of high- and low-temperature heat reservoirs, respectively; *T*_AB_ and *T*_BA_ are the interfacial temperatures between materials A and B in the ***J***_*q*_AB_ and ***J***_*q*_BA_ configurations, respectively. This calculation assumes steady-state heat flow, negligible interfacial thermal resistance, and no lateral heat loss, which allows us to get the next relations:

$$\left|J_{q\_\mathrm{AB}}\right|\;=-\frac{\int_{T_{L}}^{T_{\mathrm{AB}}}\kappa_{B}(T)\mathrm{SdT}}{L(1-x)}=-\frac{\int_{T_{\mathrm{AB}}}^{T_{H}}\kappa_{B}(T)\mathrm{SdT}}{\mathrm{Lx}}$$

$$\left|J_{q\_\mathrm{BA}}\right|\;=-\frac{\int_{T_{L}}^{T_{\mathrm{BA}}}\kappa_{A}(T)\mathrm{SdT}}{\mathrm{Lx}}=-\frac{\int_{T_{\mathrm{BA}}}^{T_{H}}\kappa_{B}(T)\mathrm{SdT}}{L(1-x)}$$

These relations and the experimentally obtained *κ*_A_(*T*) and *κ*_B_(*T*) values enable us to determine the relationship between *x* and (*T*_AB_, *T*_BA_). Consequently, the established relationship between *x* and (*T*_AB_, *T*_BA_) enables us to identify the maximum *TRR* value for any arbitrary *x*.

**Figure 3.**
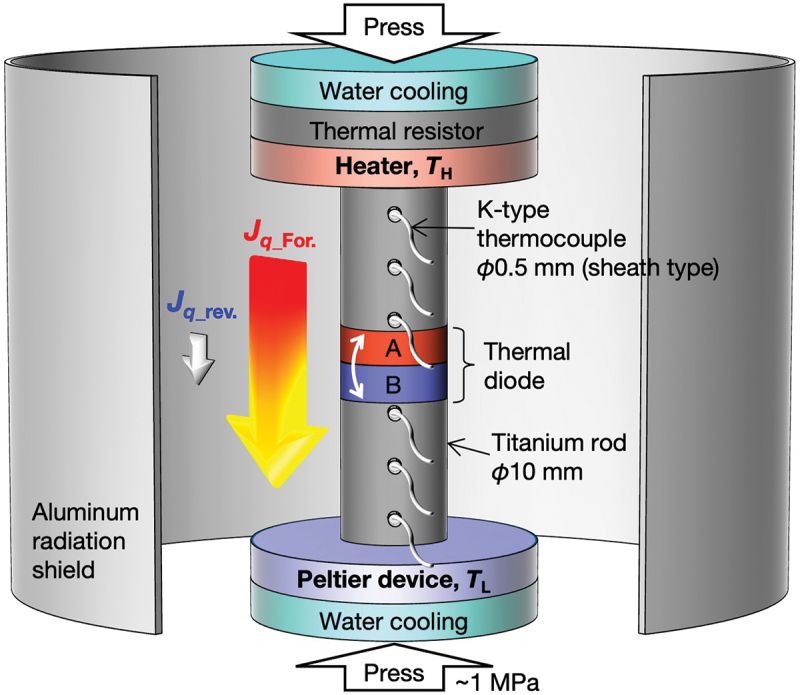
Schematic illustration of a custom-built heat flow measurement setup under steady-state conditions.

To experimentally validate the calculated thermal rectification effect, we employed a custom-built setup for measuring steady-state heat flow. As shown in Figure 3, the setup includes a heater and a Peltier device for temperature control, along with a thermal diode positioned between two titanium rods to measure heat flow. The heat flow through the thermal diode was calculated using the temperature gradient in the titanium rods and Fourier’s law. Three K-type thermocouples were inserted into the small holes drilled ~5 mm in depth in two titanium rods, the diameter and thickness of which were ~10 mm and 12 mm, respectively. The thermocouples were placed with ~3 mm intervals to monitor the temperature gradient in the titanium rods. The relatively low thermal conductivity (~20 W m^–1^ K^−1^) [21] of titanium allows us to easily analyze the temperature gradient by enhancing the temperature difference between each point of the thermometer. Additionally, the almost negligible temperature dependence of the thermal conductivity over the range of 300–900 K [21] provides us with reliability in our heat flow measurements. The experiments were conducted in a vacuum atmosphere of <10 Pa to suppress convective heat loss, and the titanium rods were enclosed with an aluminum sheet to mitigate radiative heat loss. To ensure good thermal contact, thermal grease with *κ* ~4.2 Wm^−1^K^−1^ was applied at each interface, along with a mechanical pressure of 1 MPa induced by a screw jack.

## Results

**Figure 4.**
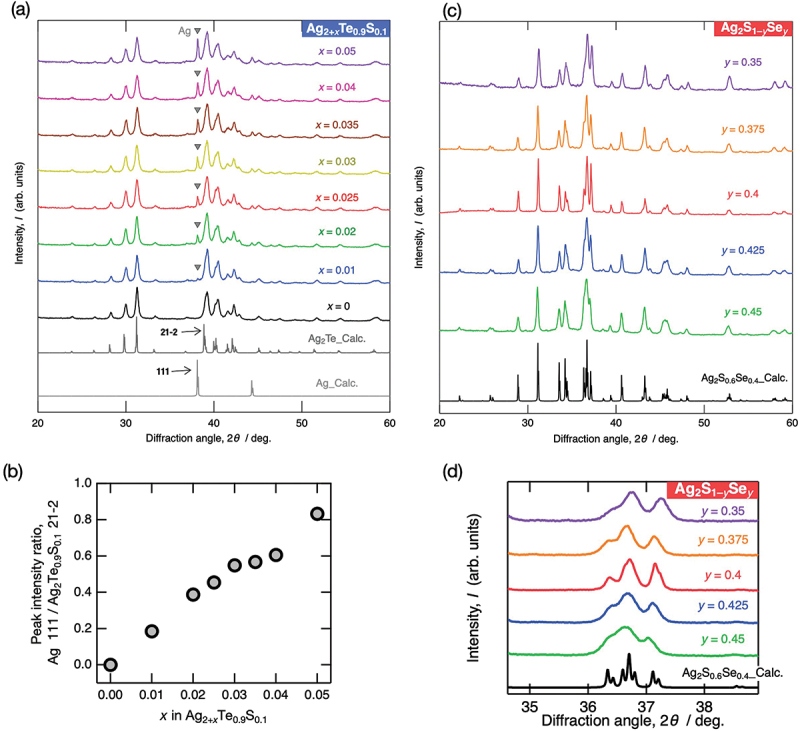
(a) bulk XRD patterns of Ag_2 + *x*_Te_0.9_S_0.1_ (*x* = 0, 0.01, 0.02, 0.025, 0.03, 0.035, 0.04, and 0.05) measured at room temperature using Cu-Kα source. (b) dependence of the peak intensity ratio, defined as Ag 111 / Ag_2_Te_0.9_S_0.1_ 21–2, on composition *x* in Ag_2 + *x*_Te_0.9_S_0.1_. (c) powder XRD patterns of Ag_2_S_1 – *y*_Se_*y*_ (*y* = 0.35, 0.375, 0.4, 0.425, and 0.45) measured at room temperature using Cu-Kα source and compared with the calculated XRD patterns of Ag_2_S_0.6_Se_0.4_ [29] using VESTA [19]. (d) magnified view of (c) around 2*θ* = 35–39°.

Figure 4(a) presents the XRD patterns of Ag_2 + *x*_Te_0.9_S_0.1_ (*x* = 0, 0.01, 0.02, 0.025, 0.03, 0.035, 0.04, and 0.05). The calculated XRD patterns of Ag_2_Te [17] and Ag [33] were obtained using VESTA [19] and plotted for comparison. The observed diffraction peaks were attributed only to the primary phase Ag_2_Te_0.9_S_0.1_ and the precipitated secondary phase of Ag. To estimate the actual change in the volume fraction of Ag impurities in the samples, the intensity ratio of the Ag 111 peak to the Ag_2_Te_0.9_S_0.1_ 21–2 peak was calculated for each composition, as shown in Figure 4(b). The intensity ratio increased consistently with increasing Ag content, confirming the proper sample preparation with the intended compositions.

Figures 4(c, d) illustrate the XRD patterns of Ag_2_S_1 – *y*_Se_*y*_ (*y* = 0.35, 0.375, 0.4, 0.425, and 0.45). As sulfur atoms were replaced by larger selenium atoms, a slight shift of the diffraction peaks toward lower angles was observed, indicating an increase in lattice constant. The success of systematic composition control in Ag_2_S_1 – *y*_Se_*y*_ was also confirmed by the systematic variation in phase transition temperature observed in the temperature-dependent thermal conductivity data presented later.

In the SEM images of Ag_2.02_Te_0.9_S_0.1_ shown in Figure S1 of the supplementary material, no Ag-enriched regions corresponding to the size of the intentionally added Ag powder (75–150 µm) were observed. This observation suggests that, during the sintering process, the Ag impurities were incorporated into HTP, exhibiting Ag superionic conduction, and subsequently precipitated in a more finely dispersed form after cooling down to LTP. Since a focused electron beam in SEM-EDX was reported to cause Ag precipitation and growth at the surface of silver chalcogenides [34], wide-scale observation was conducted here to roughly confirm the distribution of Ag impurities without causing additional Ag precipitation due to the electron beam.

**Figure 5.**
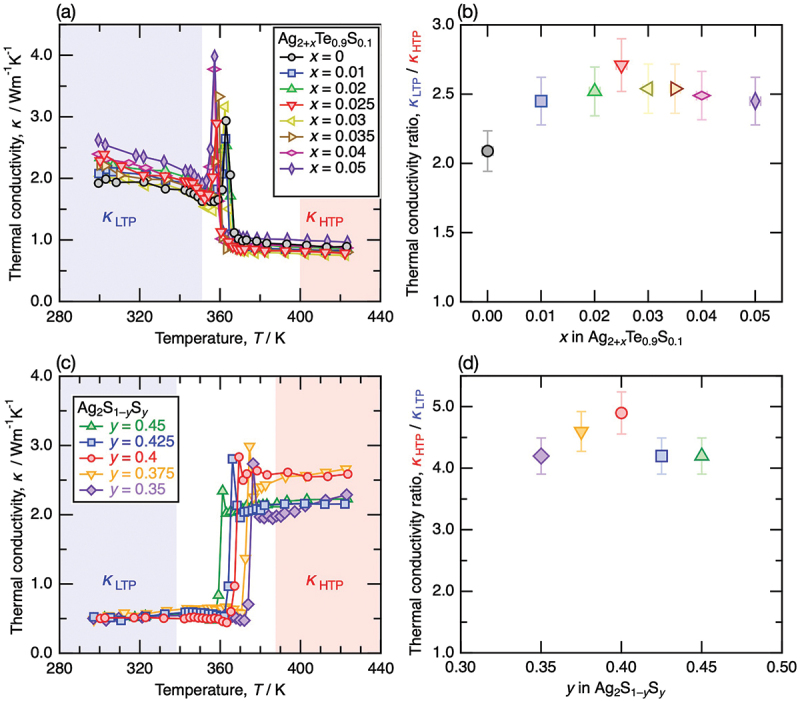
(a) temperature-dependent thermal conductivity of Ag_2 + *x*_Te_0.9_S_0.1_ (*x* = 0, 0.01, 0.02, 0.025, 0.03, 0.035, 0.04, and 0.05). (b) composition-dependent thermal conductivity ratio (*κ*_LTP_ / *κ*_HTP_) between the low-temperature phase (LTP) and the high-temperature phase (HTP) of Ag_2 + *x*_Te_0.9_S_0.1_. The thermal conductivities of the LTP and HTP were determined as the average values of the measured data in the blue and red regions in (a), respectively. (c) temperature-dependent thermal conductivity of Ag_2_S_1 – *y*_Se_*y*_ (*y* = 0.35, 0.375, 0.4, 0.425, and 0.45). (d) composition-dependent thermal conductivity ratio (*κ*_HTP_ / *κ*_LTP_) between the LTP and HTP of Ag_2_S_1 – *y*_Se_*y*_. The thermal conductivities of the LTP and HTP were determined as the average values of the measured data in the blue and red regions in (c), respectively. The uncertainty in *κ*_HTP_ / *κ*_LTP_ and *κ*_LTP_ / *κ*_HTP_ was estimated to be the same as the thermal conductivity error (~7%). Thermal conductivity peaks observed in (a) and (b) at phase transition temperatures are considered measurement artifacts in the laser flash method due to a slight discrepancy between the specific heat peak and the decrease in thermal diffusivity [35,36].

Figure 5(a) summarizes the temperature dependence of the thermal conductivity of the samples Ag_2 + *x*_Te_0.9_S_0.1_ (*x* = 0, 0.01, 0.02, 0.025, 0.03, 0.035, 0.04, and 0.05) near the phase transition temperature. The obtained composition dependence of the thermal conductivity ratio between the low-temperature phase (LTP) and the high-temperature phase (HTP), defined as*κ*_LTP_ / *κ*_HTP_, is shown in Figure 5(b). For calculating the step ratio in thermal conductivity, the magnitudes of thermal conductivities of the LTP and HTP were determined as the average of the data within the temperature ranges of *T*_LTP_ = 300–350 K and *T*_HTP_ = 400–450 K, respectively. The comparison of the thermal conductivity ratio enables a more systematic and quantitative evaluation since the measured thermal conductivity depends on sample density. For all compositions of Ag_2 + *x*_Te_0.9_S_0.1_, a significant stepwise decrease in thermal conductivity was observed during the transition from the LTP to the HTP. This reduction increased from approximately 2.1-fold at *x* = 0 to a maximum of 2.7-fold at *x* = 0.025. This result is consistent with our strategy, in which high-thermal-conductivity Ag precipitates in the LTP of Ag_2_Te_0.9_S_0.1_ and subsequently dissolves into the HTP of Ag_2_Te_0.9_S_0.1_, leading to a larger reduction in thermal conductivity across the phase transition. For *x* > 0.025, the reduction ratio of thermal conductivity gradually decreased, most likely due to the precipitation of excess Ag not only in the LTP but also in the HTP. These two effects make the reduction ratio of thermal conductivity maximum at the composition of *x* = 0.025. The intentionally added excess Ag demonstrates that LTP of Ag_2+*x*_Te_0.9_S_0.1_ exhibit enhanced thermal conductivity with increasing Ag content. This indicates that metallic Ag precipitates contribute their high electronic thermal conductivity (~400 Wm^−1^K^−1^ at room temperature [21]) to the composite rather than acting as scattering centers.

In the future, we aim to develop a system that enables in-situ high-temperature XRD on our bulk samples, in order to investigate Ag dissolution/precipitation behavior in detail. For the present study, we consider that the phase diagram reported in the literature [20,30] and our experimental observation that thermal conductivity increases in LTP and remains almost unchanged in HTP provide a reliable basis to expect Ag dissolution/precipitation associated with the phase transition.

For Ag_2_S_1 – *y*_Se_*y*_ (*y* = 0.35, 0.375, 0.4, 0.425, and 0.45), Figures 5(c, d) summarize the temperature dependence of the thermal conductivity near the phase transition temperature and the composition dependence of the thermal conductivity ratio between LTP and HTP, defined as *κ*_HTP_ / *κ*_LTP_, respectively. Here, the thermal conductivities of LTP and HTP were determined as the average of the data within the temperature ranges of *T*_LTP_ = 298–338 K and *T*_HTP_ = 388–423 K, respectively. For all compositions, a significant stepwise increase in thermal conductivity was observed during the transition from LTP to HTP. Similar to the reported phase diagrams [26], a systematic decrease in the phase transition temperature associated with increasing selenium substitution was also confirmed, providing evidence of precise sample composition control. Although this study thoroughly investigated the compositions around *y* = 0.4, which has already been reported to exhibit the largest *κ*_HTP_ / *κ*_LTP_ in the Ag_2_S_1 – *y*_Se_*y*_ system, the largest *κ*_HTP_ / *κ*_LTP_ was also observed at the same composition. The thermal conductivity of Ag_2_S_0.6_Se_0.4_ is extremely low in the LTP (~0.5 W m^−1^ K^−1^) and increases to ~ 2.5 W m^−1^ K^−1^ in the HTP, resulting in a 5-fold increase across the phase transition.

The peak in thermal conductivity observed at the phase transition temperature in several compositions is primarily attributed to the specific heat peak associated with the phase transition and is considered an artifact resulting from the laser flash measurement (a transient measurement) [35,36]. Theoretically, this contribution is suppressed by the simultaneous decrease in thermal diffusivity, and no pronounced peak should appear in thermal conductivity [35,36]. However, in some compositions, a slight discrepancy between the specific heat peak and the decrease in thermal diffusivity due to the measurement artifact resulted in an apparent peak in thermal conductivity.

**Figure 6.**
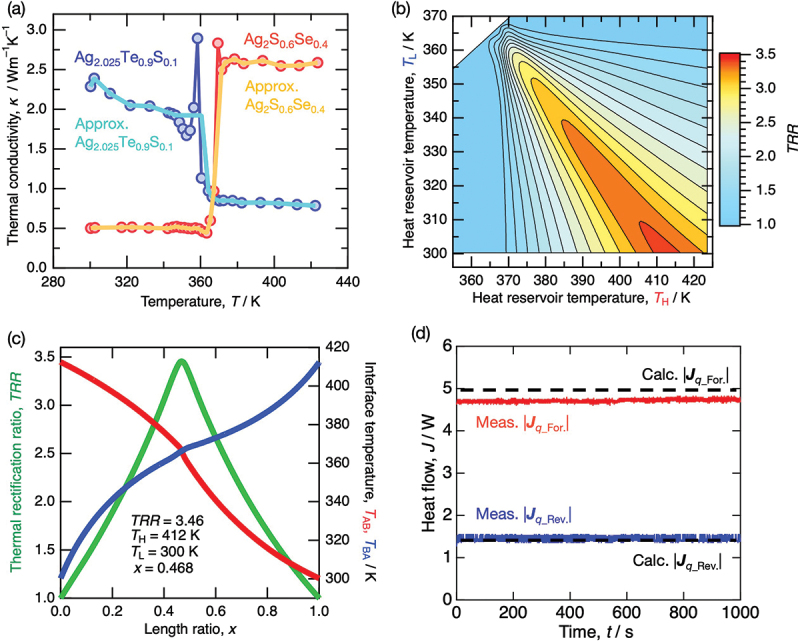
(a) temperature-dependent thermal conductivities of Ag_2.025_Te_0.9_S_0.1_ and Ag_2_S_0.6_Se_0.4_, along with the approximated data (light blue and orange solid lines, respectively) used for analysis obtained by removing the artificial peaks observed using the laser flash method. (b) contour plot showing the dependence of the maximum obtainable thermal rectification ratio (*TRR*) on the heat reservoir temperatures (*T*_H_ and *T*_L_) in the Ag_2_S_0.6_Se_0.4_-Ag_2.025_Te_0.9_S_0.1_ thermal diode. (c) dependence of interface temperatures (*T*_AB_ and *T*_BA_) and *TRR* on the length ratio x of Ag_2_S_0.6_Se_0.4_ to total length *L* in the Ag_2_S_0.6_Se_0.4_-Ag_2.025_Te_0.9_S_0.1_ thermal diode. (d) direction dependence of heat flow measured using the Ag_2_S_0.6_Se_0.4_-Ag_2.025_Te_0.9_S_0.1_ thermal diode under steady-state conditions. The red and blue solid lines represent the measured heat flows of |*J*_*q*_For._| and |*J*_*q*_Rev._|, respectively, whereas the black dashed lines represent the calculated ones.

Based on the above results, we expected the maximum thermal rectification effect using the combination of Ag_2.025_Te_0.9_S_0.1_ and Ag_2_S_0.6_Se_0.4_, which exhibited the largest stepwise decrease and increase in thermal conductivity during the phase transition, respectively. Using the temperature-dependent thermal conductivity data of Ag_2.025_Te_0.9_S_0.1_ and Ag_2_S_0.6_Se_0.4_, we calculated the maximum obtainable thermal rectification ratio (*TRR*_calc._) from a thermal diode consisting of Ag_2.025_Te_0.9_S_0.1_ and Ag_2_S_0.6_Se_0.4_ as well as the required conditions such as the corresponding heat reservoir temperatures and material length ratio [8]. Here, as shown in Figure 6(a), approximated data excluding the thermal conductivity peak during the phase transition were used in the calculations to eliminate the artifacts arising from the laser flash measurement. Figure 6(b) represents the contour plot of the calculated maximum *TRR*_calc._ obtainable from the Ag_2.025_Te_0.9_S_0.1_-Ag_2_S_0.6_Se_0.4_ thermal diode as a function of the heat reservoir temperatures (*T*_H_, *T*_L_) and the material length ratio *x*, defined as *x* = Ag_2_S_0.6_Se_0.4_ / (Ag_2_S_0.6_Se_0.4_ + Ag_2.025_Te_0.9_S_0.1_). For further detail, Figure 6(c) illustrates the dependence of *TRR* on *x* at the temperature difference of *T*_H_ = 412 K and *T*_L_ = 300 K. Consequently, we found that a large *TRR*_calc._ of 3.5 is obtainable from the Ag_2.025_Te_0.9_S_0.1_-Ag_2_S_0.6_Se_0.4_ thermal diode under the conditions of *T*_H_ = 412 K and *T*_H_ = 300 K, and a length ratio of Ag_2.025_Te_0.9_S_0.1_ : Ag_2_S_0.6_Se_0.4_ = 47:53.

o experimentally confirm the obtainable *TRR* value, we fabricated a thermal diode under the calculated conditions above and measured the directional dependence of heat flow in the steady state. Ag_2_S_0.6_Se_0.4_ and Ag_2.025_Te_0.9_S_0.1_ samples were prepared in pellet form, with the thicknesses 2.1 mm and 2.4 mm, respectively, corresponding to a length ratio of Ag_2_S_0.6_Se_0.4_ : Ag_2.025_Te_0.9_S_0.1_ = 47:53. The diameters of both samples were set to ∼ϕ10mm. These samples were bonded together using a thermal conductive grease, forming a thermal diode. The heat flow through the fabricated thermal diode was evaluated by fixing the low-temperature heat reservoir at 300 K and the high-temperature heat reservoir at 412 K. When the Ag_2_S_0.6_Se_0.4_ face was on the high-temperature side and the Ag_2.025_Te_0.9_S_0.1_ face on the low-temperature side, the observed large heat flow in the forward direction was defined as ***J***_*q*_For._, while in the reverse configuration, the observed small heat flow in the reverse direction was defined as ***J***_*q*_Rev._. Figure 6(d) plots |***J***_*q*_For._| and |***J***_*q*_Rev._| as functions of heating time. The red and blue solid lines represent the experimentally measured heat flow, whereas the dashed lines indicate the heat flow calculated based on the temperature dependence of thermal conductivity and sample dimensions. The reversal of the temperature gradient resulted in a significant thermal rectification effect being successfully observed due to the change in the magnitude of heat flow. Notably, the experimentally obtained thermal rectification ratio was *TRR*_meas._ = 3.3, exceeding the theoretical maximum *TRR* = 3 for a thermal diode made of materials with linear temperature-dependence in thermal conductivity, and representing the highest value ever reported for all-solid-state thermal diodes.

The experimentally obtained *TRR*_exp._ = 3.3 was slightly lower than the calculated value (*TRR*_calc._ = 3.5), primarily due to the deviation in the measured |***J***_*q*_For._| from the calculated one. Considering the measurement uncertainty of thermal conductivity (~7%), this difference is within a reasonable experimental error. However, it is important to note that the *TRR* calculation was performed under idealized assumptions, namely, the absence of radiative heat losses and interfacial thermal resistance. In a real experimental setup, these factors cannot be completely eliminated and thus may contribute to a reduction in the effective heat flow and, consequently, the observable thermal rectification effect. Radiative losses would reduce the measured heat flux in both the forward and reverse directions compared to the calculated values. On the other hand, interfacial thermal resistance tends to have a more pronounced effect in the forward configuration, where the total thermal resistance is lower. Indeed, it is reported that interfacial thermal resistance can significantly suppress the rectification performance in composite thermal diodes employing high-thermal-conductivity materials [7,37]. In this study, the constituent materials exhibit relatively low thermal conductivity (0.5–2.5 Wm^−1^K^−1^), and the effect of interfacial thermal resistance is expected to have a minor impact. However, since the phase transition temperatures of the used materials are nearly identical, even slight deviations in the interface temperature from the ideal condition could be a non-negligible introduction of error, suggesting that the obtained error in our experiment is more likely attributed to the influence of interfacial thermal resistance.

## Discussion

Under the experimental conditions in this study (duration of 1000 s and a temperature gradient of Δ*T* = 112 K), thermal stability was confirmed without any significant degradation in rectification performance. However, under extended durations and larger temperature gradients beyond these conditions, the high ionic mobility of Ag^+^ in HTP (superionic conduction nature of silver chalcogenides) may lead to Ag precipitation, potentially degrading thermal performance. This concern is consistent with prior studies on superionic conduction behavior under temperature gradient in the field of thermoelectrics [38,39]. In the thermoelectric field, similar challenges have been encountered in Cu-based chalcogenides, where Cu^+^ migration leads to time-dependent degradation, and strategies such as interstitial-site atomic doping and advanced electrode design have been employed to suppress Cu precipitation [38,39]. These approaches may also be effective for mitigating Ag migration in silver chalcogenide-based thermal diodes, representing an important direction for future improvement.

In all-solid-state thermal diodes utilizing structural phase transition materials, the durability of the constituent materials under repeated phase transitions accompanied by volume changes due to temperature cycling requires careful consideration. Ag_2_S_1–*y*_Se_*y*_ (*y* ≤ 0.6), which exhibits an Ag_2_S-type crystal structure, has been reported to possess metal-like deformability and mechanical compliance, suggesting that these materials have high stability against volume changes in the phase transition cycles [40,41]. On the other hand, Ag_2_Te_1–*x*_S_*x*_ (*x* ≤ 0.15) compounds experience relatively large volume changes across the phase transition, possibly making them more brittle compared to Ag_2_S_1 – *y*_Se [41]. However, the incorporated Ag impurities in Ag_2+*δ*_Te_1–*x*_S_*x*_ in this study may also enhance the durability of the material by acting as metallic adhesives. This effect was not directly evaluated here and was just presented as a hypothesis, supported by prior studies on metal-matrix composites [42,43].

To achieve a larger thermal rectification effect beyond this study, we need to identify materials with even larger reductions in thermal conductivity across the phase transition than that of Ag_2.025_Te_0.9_S_0.1_. For the enhanced thermal conductivity variations across the phase transition, one of the key requirements is to explore materials with greater variations in electronic thermal conductivity and moderately low lattice thermal conductivity. For example, Cu_2.9_Te_2_ has been reported to exhibit a significant stepwise decrease in thermal conductivity, reaching a *κ*_LTP_ / *κ*_HTP_ ~5–6 across the phase transition temperature (*T*_*x*_ ~650 K) [44]. By tuning the phase transition temperature within the 360–380 K range in the same manner as Ag_2_S_1–*y*_Se_*y*_ (*y* ~0.4) presumably through the chalcogenide-site substitution, it would be possible to fabricate thermal diodes showing much higher thermal rectification ratios of *TRR* ≥4.

## Conclusion

To develop an all-solid-state composite thermal diode with a thermal rectification ratio (*TRR*) exceeding 3, which is the theoretical maximum with materials possessing linear temperature-dependent thermal conductivity, we synthesized various compositions of silver chalcogenides, Ag_2 + *x*_Te_0.9_S_0.1_ (*x* = 0, 0.01, 0.02, 0.025, 0.03, 0.035, 0.04, and 0.05) and Ag_2_S_1 – *y*_Se_*y*_ (*y* = 0.35, 0.375, 0.4, 0.425, and 0.45), and investigated their temperature-dependent thermal conductivities and composition-dependent stepwise thermal conductivity changes associated with the phase transition in detail. For Ag_2 + *x*_Te_0.9_S_0.1_, high thermal conductivity silver impurities were intentionally introduced to precipitate in the low-temperature phase and to dissolve into the high-temperature phase of Ag₂Te_0.9_S_0.1_ matrix, resulting in the largest step-down behavior of 2.7-fold at the composition of *x* = 0.025. On the other hand, Ag_2_S_1 – *y*_Se_*y*_ (*y* = 0.35, 0.375, 0.4, 0.425, and 0.45), showed the largest stepwise increase in thermal conductivity at *y* = 0.4, reaching a maximum increase of 5-fold. We then fabricated a thermal diode consisting of Ag_2.025_Te_0.9_S_0.1_ and Ag_2_S_0.6_Se_0.4_ at the length ratio of Ag_2.025_Te_0.9_S_0.1_:Ag_2_S_0.6_Se_0.4_ = 53:47 and measured the direction-dependent heat flow to estimate thermal rectification ratio. When the thermal diode was placed between the heat reservoirs kept at *T*_L_ = 300 K and *T*_H_ = 412 K, we observed a *TRR* of 3.3, which is the highest value reported for an all-solid-state composite thermal diode. The demonstrated strategy, in which the precipitation/dissolution of high-thermal-conductivity impurities enhances the stepwise decrease in thermal conductivity during the phase transition, would lead to the development of new high-performance thermal diodes, thermal switches, and other thermal management devices.

## Supplementary Material

Supplemental Material

## Data Availability

The data that support the findings of this study are available from the corresponding author upon reasonable request.
