# Supplementary material for: Enhanced rectification effect in silver chalcogenide-based thermal diode by using precipitation/dissolution of Ag impurity across the structure phase transition
Source: Sci Technol Adv Mater. 2025 Aug 28;26(1):2549674. doi: 10.1080/14686996.2025.2549674 (PMC12447454; doi:10.1080/14686996.2025.2549674)
Supplement: Supplemental Material [file TSTA_A_2549674_SM3485.docx]

**Supplementary Material**

**for**

**Enhanced rectification effect in silver chalcogenide-based thermal diode**

**by using precipitation/dissolution of Ag impurity across**

**the structure phase transition**

Keisuke Hirata ^a, b^, Yusuke Goto ^a^, and Tsunehiro Takeuchi ^a, b, *^

*^a^ Toyota Technological Institute, Nagoya, Aichi 468-8511, Japan.*

*^b^ Research Center for Smart Energy Technology of Toyota Technological Institute, Nagoya, Aichi 468-8511, Japan.*

*^*^ Corresponding author: t_takeuchi@toyota-ti.ac.jp*

**Contents**

1. **SEM-EDX observation of Ag_2.02_Te_0.9_S_0.1_**
2. **SEM-EDX observation of Ag_2.02_Te_0.9_S_0.1_**


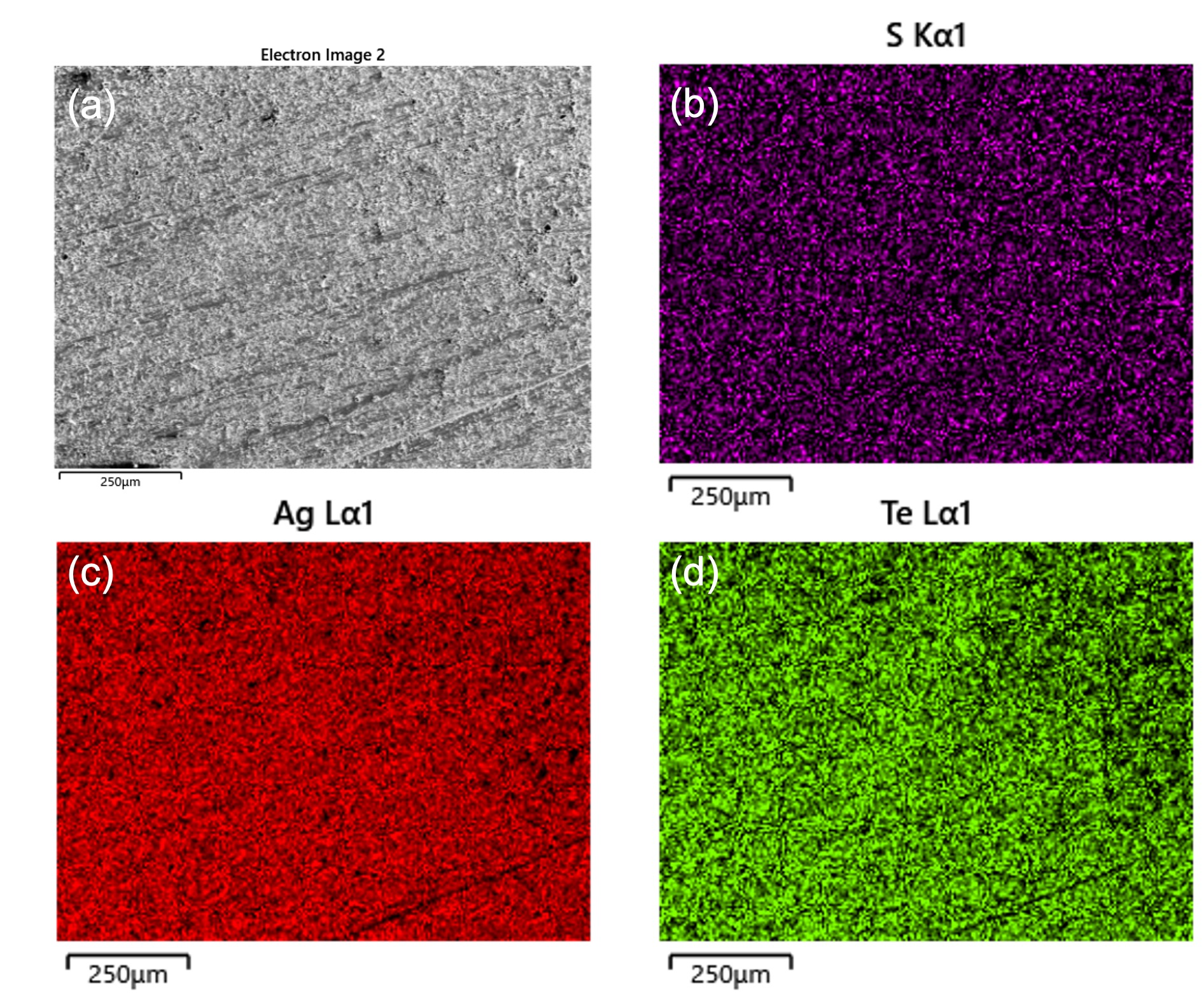


Figure S1. SEM-EDX observation for Ag_2.02_Te_0.9_S_0.1_ bulk surface: **(a)** secondary electron imaging and **(b-d)** elemental distribution mapping for S, Ag, and Te.
